# Mapping the Mechanisms of Transcranial Alternating Current Stimulation: A Pathway from Network Effects to Cognition

**DOI:** 10.3389/fpsyt.2014.00162

**Published:** 2014-11-20

**Authors:** Ruairidh M. Battleday, Timothy Muller, Michael S. Clayton, Roi Cohen Kadosh

**Affiliations:** ^1^Department of Experimental Psychology, University of Oxford, Oxford, UK

**Keywords:** neuroenhancement, transcranial electrical stimulation, transcranial alternating current stimulation, non-invasive brain stimulation, cognitive enhancement, oscillations, oscillatory activity

## Introduction

In recent decades, our appreciation of the complexity of the brain has deepened immensely, as has our understanding of how it performs key functions. In the face of such complexity, and given the rising cost of neuropsychiatric illness (1), an intriguing question is whether we can promote further understanding, and in some cases enhancement, of the typical and atypical brain by targeted modulation of its activity. Notably, transcranial alternating current stimulation (tACS) – which involves transcranial application of weak sinusoidal electrical currents (2) – seems ideally suited to address this question, as it has been demonstrated to modulate endogenous oscillatory electrical activity (3), enhance cognitive functions (4–7), and provide support in neurological disease (8, 9). However, a complete mechanistic pathway between the neuronal and cognitive effects of tACS remains in need of explication, precluding both significant theoretical contribution by tACS studies, and the development of more adaptive neuroenhancement regimes. Therefore, in this Opinion article, we briefly review the role of oscillatory neuronal activity in cognition, before outlining one potential pathway by which the interaction between tACS and endogenous oscillations at a network level may be reconciled with its effects on broader cognitive functions.

## The Roles of Cortical Oscillations in Cognition

**Figure 1:**
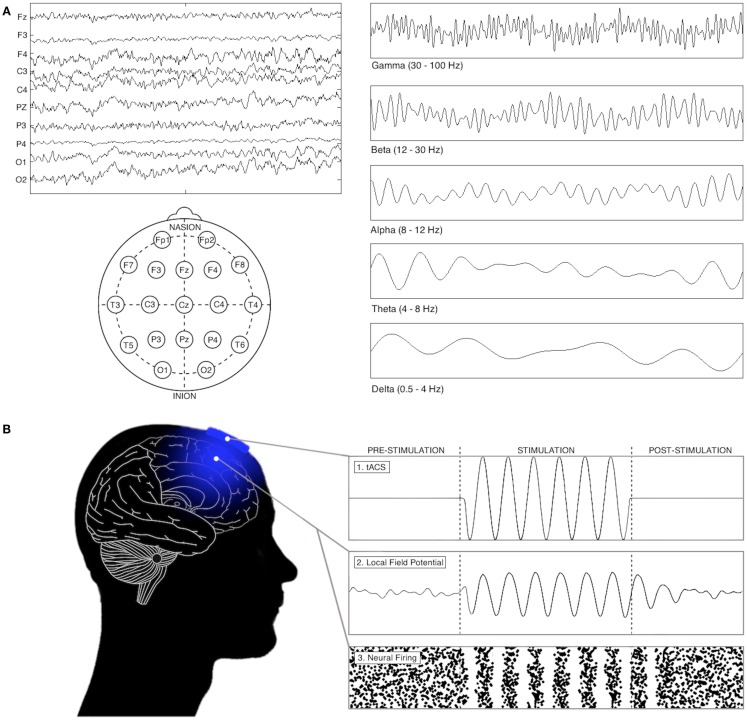
**Oscillations in electrophysiological recordings and the effects of tACS on network activity**. **(A)** Electrophysiological recordings can reveal important information about the brain, in both resting and task-oriented states. Left: the electroencephalogram, which displays electrical signals from a series of electrodes placed on the scalp. Right: when electrophysiological recordings are filtered oscillatory patterns emerge in a task-, area-, and state-dependent manner, typically divided into delta (0.5–4 Hz), theta (4–7 Hz), alpha (7–12 Hz), beta (12–30 Hz), and gamma (30–100+ Hz) frequency bands. **(B)** The interaction between tACS and neural firing (shown through both local field potential fluctuations and changes in neural firing patterns). In animals, theoretical, and human work, tACS has been found to increase the power of oscillations and cause them to synchronize their fluctuations with incoming stimulation (see text).

Synchronous oscillations in the firing rates of large populations of neurons – recorded as fluctuations in electrical field potentials – represent a highly organized form of brain activity (see Figure 1). Specific oscillatory frequencies emerge in a task-, area-, and state-dependent manner, and are thought to reflect structural and functional features of the active neuronal networks mediating local and long-range cortical functions, and their cognitive manifestations [see in Ref. (10)]. For example, alpha oscillations (8–12 Hz) over occipito-parietal cortices have been suggested to facilitate the inhibition of task-irrelevant visual processing (11), while increased oscillatory synchronization between frontal and parietal cortices in multiple frequency bands is thought critical to the orientation of attention (12) and the maintenance of working memory (13). Importantly, reliable alterations in oscillatory activity also feature in the pathophysiology of several neurological and psychiatric conditions (14–17).

## The Interaction between tACS and Endogenous Oscillations

Although the currents applied in tACS reliably generate fluctuations in individual neuronal firing rates (18), their interaction with larger ensembles in humans remains less clear. Nevertheless, two mechanistic insights have been derived from animal, computational, and human behavioral studies [see in Ref. (18, 19)]: (1) that in certain situations tACS may lock endogenous oscillatory activity to its frequency and phase (“entrainment”; see Figure 1); and, (2) that it may amplify a network’s activity by stimulation at its “natural” frequency (“resonance”). However, the extent to which the behavioral and electrophysiological effects of tACS depend on these interactions remains unclear. As clarifying this issue is critical for full mechanistic understanding of tACS, and due to the recent publication of several studies that inform this debate, we review these theories below, before attempting to identify sufficient commonality between them to allow a more mechanistic consideration of the effects of tACS on cognition.

Entrainment refers to the synchronization of the frequency and phase of neuronal activity to external stimuli, such as rhythmic visual stimulation (20), or activity in other brain modules. In support of the theory that tACS entrains endogenous activity, weak alternating currents have been shown to shift the frequency and phase of neuronal firing *in vivo, in vitro*, and in computational models (18, 21–25). According to these computational accounts, entrainment increases the number of individual neurons firing at the peak of a particular oscillation – and therefore its power – in a manner that could conceivably affect behavior (18, 23). This theory has been corroborated by two recent human studies, the first of which reported increases in *endogenous* parieto-occipital alpha power during alpha-tACS to the same regions, noting that specific phases of tACS correlated with improved accuracy on a visual oddball task (3). The second reported that induction of lucid dreaming occurred with increases in *endogenous* fronto-temporal activity at 25 and 40 Hz during 25 and 40 Hz stimulation, respectively (26). Indirect illustrations of the benefits of artificially entraining two endogenous oscillating systems have also been provided: Polania and colleagues reported decreased reaction times on a working memory task during 6 Hz tACS to left prefrontal and parietal cortices with 0° phase alignment (“phasic”), and increased reaction times during tACS with 180° phase alignment (“anti-phasic”) (4). Similarly, Strüber and colleagues reported that anti-phasic 40 Hz tACS delivered to both parieto-occipital cortices caused perceptual alteration, whereas phasic tACS did not (27).

Two things are striking about the majority of tACS studies, including those mentioned above: (1) behavioral and electrophysiological amplification is typically only elicited at a single stimulation frequency; and, (2) this tends to be the frequency that dominates electrophysiological recordings of unstimulated subjects completing similar tasks, during *similar behavioral states*. For example, Feurra and colleagues showed that beta tACS of the motor cortex – which oscillates at beta frequencies during rest – but *not* stimulation at other frequencies, or to other areas, directly influenced motor cortex electrophysiology (28). Further, groups have shown that in the somatosensory (29) and visual (30) systems, tACS only elicits behavioral effects at frequencies similar to those recorded from the cortex during analogous sensory stimulation. Although it was initially argued that effects reported in the visual paradigm – namely, that phosphene perception was induced by alpha-tACS to the visual cortex in the dark, and beta (12–30 Hz) in the light, the dominant oscillations recorded in this area in dark and light environments, respectively – could instead result from retinal activation (31–33), Kanai and colleagues subsequently showed that beta tACS of occipital *but not frontal* cortex directly interacts with underlying neuronal activity (34), casting doubt on the contribution of any volume conduction effect. Thus, it would seem that – rather than *shifting* the frequency and phase of endogenous oscillations – the causal interaction between tACS and neuronal networks depends on *matching* stimulation frequency with endogenous activity. According to the phenomenon of resonance, if task-activated oscillatory brain networks were stimulated at their resting frequency this could lead to augmentation of their activity (35). In support of this theory, Rosanova and colleagues showed that cortico-thalamic modules resonate at distinct frequencies if perturbed using transcranial magnetic stimulation (namely, alpha-band in occipital cortex, beta-band in parietal, and fast-beta-/gamma-band in frontal) (36). Further, Schmidt and colleagues showed that applying low intensity alternating electrical fields to an optogenetically induced “endogenous” 1 Hz firing rhythm in mouse neocortical slices preferentially increased oscillatory activity at 1 Hz and its first harmonic (2 Hz), collaborating model-predicted tACS-induced resonance phenomena (22, 37). Interestingly, stimulation using higher currents abolished this selectivity, implying that the ability of stimulation to drive network activity may be less constrained by the natural frequency of an area at higher intensities (38).

Although the entrainment and resonance accounts appear discrete, a common theme can be identified between them: the most important network effect of tACS is the wider recruitment of a – presumably previously partially enlisted – population of neurons into task-relevant rhythmically firing networks, occurring as a corollary of tightened synchrony or resonance properties. In this context, phase alignment between stimulation and endogenous oscillations is still important, as stipulated by theoretical accounts of entrainment (23) and resonance (39), and demonstrated by the human behavioral studies discussed above (3, 4, 26, 27). Thus, we propose that – at the low intensities applied using tACS – matching stimulation and endogenous frequencies are a necessary condition to enable the causal amplification of task-specific network activity, likely through an interaction that involves entrainment *and* resonance. This matching may proceed gradually, via entrainment, or may be imparted based on concurrent electrophysiology.

## Moving from Network Effects to Cognitive Functions

Further investigation of the network effects of tACS is clearly required; however, the question of how recruitment of a larger population of neurons oscillating at a particular frequency might affect a nebulous function like cognition may still be approached. To do so succinctly, the following premises must first be accepted: that particular cognitive functions are achieved by the activity of distributed brain networks; that particular cortical regions perform sub-computations of these functions; and that oscillations within brain tissue selectively enhance transfer of information through this network [see in Ref. (10, 40)].

Since tACS targets a relatively localized cortical region, causing more widespread enlistment into a task- and state-activated rhythm should have direct consequences on information processing by that area. Neurons are more likely to fire in response to each other if they are synchronized, as input is more likely to be transmitted at periods of mutual depolarization (41). Thus, it is possible that tACS-induced increases in local coherent activity enhance information transfer and processing within the subset of oscillating networks that subserve task functions, thereby altering their contribution to cognitive processes. For example, given the hypothesized role of alpha rhythms in irrelevant visual input suppression (11), increasing the power of alpha oscillations could enhance the suppression of task-irrelevant visual inputs, and improve overall performance on visual attention tasks. This could conceivably have occurred in Helfrich and colleagues’ study, where alpha-tACS increased alpha power over parieto-occipital cortices and improved performance in a visual task (3). Equally, if the inhibitory–excitatory balance or physical orientation of enlisted neurons were to differ markedly from the already active neuronal population, they could alter computations performed by that network; indeed, the sensitivity of neurons to applied electrical fields appears dependent on whether they are inhibitory or excitatory (42), and their orientation and geometry (43, 44). In this context, Brignani and colleagues’ report of *deterioration* of visual performance following alpha or theta tACS to visual cortex regardless which hemisphere was stimulated warrants further investigation, as it questions the degree to which the effects of tACS can be considered local or frequency-specific (45).

As it is now thought that distributed cortico-thalamic networks mediate the computations underlying broad cognitive functions (46), it follows that tACS could also engender cognitive enhancement through modification of an area’s function within such a network. Such an interaction could explain the reduction in the time taken to solve Raven’s Matrices – a test of fluid intelligence, a function purportedly mediated by a *multi*-region network (47) – following gamma tACS of the left frontal lobe (in this case 40 Hz) (6). According to Fries’s communication-through-coherence hypothesis, information is passed more efficiently between network components if they are “coupled” to each other through synchronous depolarizations. Coupling could be established by increased synchronization of a region’s output onto a recipient area, which would increase the impact of its output due to the time dependence of post-synaptic summation, and if sufficient lead to entrainment (40). It follows that by increasing synchrony in one area, tACS could affect its coupling with other network components, and thereby alter the cognitive function that emerges from the activity of that network. Notably, this theory predicts the results of Polanía and colleagues’ study, in which two areas were artificially coupled at phasic and anti-phasic alignments (4). Finally, amplification of oscillatory power could alter synaptic weighting within the components of a network, potentially in turn augmenting and sustaining direct amplification of oscillatory activity (48).

## Discussion

In summary, by virtue of entrainment and resonance, tACS may modulate cognitive functions by enlisting a wider population of neurons into a local oscillating network, and in turn alter both the internal computations performed by an area and activity within a wider cerebral network. A critical question remains whether tACS can only be used to modulate one network per area per task. If this is the case, future improvements of tACS will depend on cataloging the spatio-temporal evolution of oscillatory patterns during task completion, as well as improving technology for applying tACS in single and multiple areas (49). If, instead, a number of networks may be selectively enlisted by stimulation at their distinct frequencies, the potential for probing and enhancing cerebral network function is vast. In either case, striving for a full mechanistic account of tACS remains vital in enabling tACS to be applied with maximal efficacy, in healthy and ill populations alike.

## Conflict of Interest Statement

The authors declare that the research was conducted in the absence of any commercial or financial relationships that could be construed as a potential conflict of interest.

## References

[1] Insel TR Assessing the economic costs of serious mental illness. Am J Psychiatry (2008) 165:663–5 10.1176/appi.ajp.2008.08030366 18519528

[2] Antal A Boros K Poreisz C Chaieb L Terney D Paulus W. Comparatively weak after-effects of transcranial alternating current stimulation (tACS) on cortical excitability in humans. Brain Stimul (2008) 1:97–105. 10.1016/j.brs.2007.10.001 20633376

[3] Helfrich RF Schneider TR Rach S Trautmann-Lengsfeld SA Engel AK Herrmann CS. Entrainment of brain oscillations by transcranial alternating current stimulation. Curr Biol (2014) 24:333–9. 10.1016/j.cub.2013.12.041 24461998

[4] Polanía R Nitsche MA Korman C Batsikadze G Paulus W. The importance of timing in segregated theta phase-coupling for cognitive performance. Curr Biol (2012) 22:1314–8. 10.1016/j.cub.2012.05.021 22683259

[5] Pahor A Jaušovec N. The effects of theta transcranial alternating current stimulation (tACS) on fluid intelligence. Int J Psychophysiol (2014) 93:322–31. 10.1016/j.ijpsycho.2014.06.015 24998643

[6] Santarnecchi E Polizzotto NR Godone M Giovannelli F Feurra M Matzen L Frequency-dependent enhancement of fluid intelligence induced by transcranial oscillatory potentials. Curr Biol (2013) 23:1449–53. 10.1016/j.cub.2013.06.022 23891115

[7] Kar K Krekelberg B. Transcranial alternating current stimulation attenuates visual motion adaptation. J Neurosci (2014) 34:7334–40. 10.1523/JNEUROSCI.5248-13.2014 24849365 PMC4028503

[8] Brittain J-S Probert-Smith P Aziz TZ Brown P. Tremor suppression by rhythmic transcranial current stimulation. Curr Biol (2013) 23:436–40. 10.1016/j.cub.2013.01.068 23416101 PMC3629558

[9] Fedorov A Chibisova Y Szymaszek A Alexandrov M Gall C Sabel BA. Non-invasive alternating current stimulation induces recovery from stroke. Restor Neurol Neurosci (2010) 28:825–33. 10.3233/RNN-2010-0580 21209497

[10] Wang X-J. Neurophysiological and computational principles of cortical rhythms in cognition. Physiol Rev (2010) 90:1195–268. 10.1152/physrev.00035.2008 20664082 PMC2923921

[11] Jensen OMA. Shaping functional architecture by oscillatory alpha activity: gating by inhibition. Front Hum Neurosci (2010) 4:186. 10.3389/fnhum.2010.00186 21119777 PMC2990626

[12] Daitch AL Sharma M Roland JL Astafiev SV Bundy DT Gaona CM Frequency-specific mechanism links human brain networks for spatial attention. Proc Natl Acad Sci U S A (2013) 110(48):19585–90. 10.1073/pnas.1307947110 24218604 PMC3845177

[13] Salazar RF Dotson NM Bressler SL Gray CM. Content-specific fronto-parietal synchronization during visual working memory. Science (2012) 338:1097–100. 10.1126/science.1224000 23118014 PMC4038369

[14] Brown P Oliviero A Mazzone P Insola A Tonali P Di Lazzaro V. Dopamine dependency of oscillations between subthalamic nucleus and pallidum in Parkinson’s disease. J Neurosci (2001) 21:1033–8. 11157088 10.1523/JNEUROSCI.21-03-01033.2001 PMC6762327

[15] Worrell GA Parish L Cranstoun SD Jonas R Baltuch G Litt B. High-frequency oscillations and seizure generation in neocortical epilepsy. Brain (2004) 127:1496–506. 10.1093/brain/awh149 15155522

[16] Montez T Poil S-S Jones BF Manshanden I Verbunt JP van Dijk BW Altered temporal correlations in parietal alpha and prefrontal theta oscillations in early-stage Alzheimer disease. Proc Natl Acad Sci U S A (2009) 106:1614–9. 10.1073/pnas.0811699106 19164579 PMC2635782

[17] Uhlhaas PJ Singer W. Abnormal neural oscillations and synchrony in schizophrenia. Nat Rev Neurosci (2010) 11:100–13. 10.1038/nrn2774 20087360

[18] Reato D Rahman A Bikson M Parra LC. Low-intensity electrical stimulation affects network dynamics by modulating population rate and spike timing. J Neurosci (2010) 30:15067–79. 10.1523/JNEUROSCI.2059-10.2010 21068312 PMC3500391

[19] Reato D Rahman A Bikson M Parra LC. Effects of weak transcranial alternating current stimulation on brain activity-a review of known mechanisms from animal studies. Front Hum Neurosci (2013) 7:687. 10.3389/fnhum.2013.00687 24167483 PMC3805939

[20] De Graaf TA Gross J Paterson G Rusch T Sack AT Thut G. Alpha-band rhythms in visual task performance: phase-locking by rhythmic sensory stimulation. PLoS One (2013) 8:e60035. 10.1371/journal.pone.0060035 23555873 PMC3612058

[21] Fröhlich F McCormick DA. Endogenous electric fields may guide neocortical network activity. Neuron (2010) 67:129–43. 10.1016/j.neuron.2010.06.005 20624597 PMC3139922

[22] Ali MM Sellers KK Fröhlich F. Transcranial alternating current stimulation modulates large-scale cortical network activity by network resonance. J Neurosci (2013) 33:11262–75. 10.1523/JNEUROSCI.5867-12.2013 23825429 PMC6618612

[23] Radman T Su Y An JH Parra LC Bikson M. Spike timing amplifies the effect of electric fields on neurons: implications for endogenous field effects. J Neurosci (2007) 27:3030–6. 10.1523/JNEUROSCI.0095-07.2007 17360926 PMC6672570

[24] Ozen S Sirota A Belluscio MA Anastassiou CA Stark E Koch C Transcranial electric stimulation entrains cortical neuronal populations in rats. J Neurosci (2010) 30:11476–85. 10.1523/JNEUROSCI.5252-09.2010 20739569 PMC2937280

[25] Deans JK Powell AD Jefferys JGR. Sensitivity of coherent oscillations in rat hippocampus to AC electric fields. J Physiol (2007) 583:555–65. 10.1113/jphysiol.2007.137711 17599962 PMC2277040

[26] Voss U Holzmann R Hobson A Paulus W Koppehele-Gossel J Klimke A Induction of self awareness in dreams through frontal low current stimulation of gamma activity. Nat Neurosci (2014) 17:810–2. 10.1038/nn.3719 24816141

[27] Strüber D Rach S Trautmann-Lengsfeld SA Engel AK Herrmann CS. Antiphasic 40 Hz oscillatory current stimulation affects bistable motion perception. Brain Topogr (2014) 27:158–71. 10.1007/s10548-013-0294-x 23709044

[28] Feurra M Bianco G Santarnecchi E Del Testa M Rossi A Rossi S. Frequency-dependent tuning of the human motor system induced by transcranial oscillatory potentials. J Neurosci (2011) 31:12165–70. 10.1523/JNEUROSCI.0978-11.2011 21865459 PMC6623220

[29] Feurra M Paulus W Walsh V Kanai R Frequency specific modulation of human somatosensory cortex. Front Psychol (2011) 2:13 10.3389/fpsyg.2011.00013 21713181 PMC3111335

[30] Kanai R Chaieb L Antal A Walsh V Paulus W Frequency-dependent electrical stimulation of the visual cortex. Curr Biol (2008) 18:1839–43 10.1016/j.cub.2008.10.027 19026538

[31] Schutter DJLG Hortensius R. Retinal origin of phosphenes to transcranial alternating current stimulation. Clin Neurophysiol (2010) 121:1080–4. 10.1016/j.clinph.2009.10.038 20188625

[32] Schwiedrzik CM. Retina or visual cortex? The site of phosphene induction by transcranial alternating current stimulation. Front Integr Neurosci (2009) 3:6. 10.3389/neuro.07.006.2009 19506706 PMC2691656

[33] Terhune DB Song SM Cohen Kadosh R Transcranial alternating current stimulation reveals atypical 40 Hz phosphene thresholds in synaesthesia. Cortex (2015) 63:267–70 10.1016/j.cortex.2014.09.006 25303273

[34] Kanai R Paulus W Walsh V. Transcranial alternating current stimulation (tACS) modulates cortical excitability as assessed by TMS-induced phosphene thresholds. Clin Neurophysiol (2010) 121:1551–4. 10.1016/j.clinph.2010.03.022 20382069

[35] Buzsaki G Rhythms of the Brain. New York: Oxford University Press (2006).

[36] Rosanova M Casali A Bellina V Resta F Mariotti M Massimini M. Natural frequencies of human corticothalamic circuits. J Neurosci (2009) 29:7679–85. 10.1523/JNEUROSCI.0445-09.2009 19535579 PMC6665626

[37] Merlet I Birot G Salvador R Molaee-Ardekani B Mekonnen A Soria-Frish A From oscillatory transcranial current stimulation to scalp EEG changes: a biophysical and physiological modeling study. PLoS One (2013) 8:e57330. 10.1371/journal.pone.0057330 23468970 PMC3585369

[38] Schmidt SL Iyengar AK Foulser AA Boyle MR Fröhlich F. Endogenous cortical oscillations constrain neuromodulation by weak electric fields. Brain Stimul (2014). 10.1016/j.brs.2014.07.033 25129402 PMC4259839

[39] Longtin A Stochastic resonance in neuron models. J Stat Phys (1993) 70:309–27 10.1007/BF01053970

[40] Fries P. A mechanism for cognitive dynamics: neuronal communication through neuronal coherence. Trends Cogn Sci (2005) 9:474–80. 10.1016/j.tics.2005.08.011 16150631

[41] Azouz R Gray CM. Adaptive coincidence detection and dynamic gain control in visual cortical neurons in vivo. Neuron (2003) 37:513–23. 10.1016/S0896-6273(02)01186-8 12575957

[42] Moliadze V Atalay D Antal A Paulus W. Close to threshold transcranial electrical stimulation preferentially activates inhibitory networks before switching to excitation with higher intensities. Brain Stimul (2012) 5:505–11. 10.1016/j.brs.2011.11.004 22445135

[43] Bikson M Inoue M Akiyama H Deans JK Fox JE Miyakawa H Effects of uniform extracellular DC electric fields on excitability in rat hippocampal slices in vitro. J Physiol (2004) 557:175–90. 10.1113/jphysiol.2003.055772 14978199 PMC1665051

[44] Miranda PC Mekonnen A Salvador R Ruffini G. The electric field in the cortex during transcranial current stimulation. Neuroimage (2013) 70:48–58. 10.1016/j.neuroimage.2012.12.034 23274187

[45] Brignani D Ruzzoli M Mauri P Miniussi C. Is transcranial alternating current stimulation effective in modulating brain oscillations? PLoS One (2013) 8:e56589. 10.1371/journal.pone.0056589 23457586 PMC3573000

[46] Sporns O Chialvo DR Kaiser M Hilgetag CC Organization, development and function of complex brain networks. Trends Cogn Sci (2004) 8:418–25 10.1016/j.tics.2004.07.008 15350243

[47] Jung RE Haier RJ. The parieto-frontal integration theory (P-FIT) of intelligence: converging neuroimaging evidence. Behav Brain Sci (2007) 30:135–54. 10.1017/S0140525X07001185 17655784

[48] Zaehle T Rach S Herrmann CS. Transcranial alternating current stimulation enhances individual alpha activity in human EEG. PLoS One (2010) 5:e13766. 10.1371/journal.pone.0013766 21072168 PMC2967471

[49] Fröhlich F. Endogenous and exogenous electric fields as modifiers of brain activity: rational design of noninvasive brain stimulation with transcranial alternating current stimulation. Dialogues Clin Neurosci (2014) 16:93–102. 24733974 10.31887/DCNS.2014.16.1/ffroehlich PMC3984895

